# Sex differences in patterns of relations between family interactions and depressive symptoms in adolescents

**DOI:** 10.3325/cmj.2011.52.469

**Published:** 2011-08

**Authors:** Sanja Smojver-Ažić, Petar Bezinović

**Affiliations:** 1 Department of Psychology, Faculty of Humanities and Social Sciences, University of Rijeka, Rijeka, Croatia; 2 Institute for Social Research in Zagreb, Zagreb, Croatia

## Abstract

**Aim:**

To gain insight into the relations between protective/risk family interactions and depressive symptoms in adolescent boys and girls.

**Method:**

A self-reported cross-sectional survey was conducted on a representative sample of 1191 secondary school students (617 girls and 574 boys) aged from 14 to 19 years, with a median of 16, from all secondary schools in the Primorsko-goranska County, Croatia in January and February 2010. Students reported their depressive symptoms, perceptions about the relationship with their mother and father, family activities, and parents’ conflict resolution strategies. Data were analyzed by hierarchical multiple regression to calculate the effects of family supportive and harmful interactions on depressive symptoms in girls and boys.

**Results:**

Depressive symptoms were reported often and very often by 19.1% of girls and 15.8% of boys. Girls’ assessment of the family relations was significantly more positive than boys’, including the assessment of family activities, constructive family conflict resolution, or father’s and mother’s warmth and affection. Multiple correlation analysis revealed that the examined family variables accounted for 16.3% of the variance of depressive symptoms in boys and for 17.2% in girls. Hierarchical multiple regression analysis showed a difference in the relation of family variables and depressive symptoms between boys and girls. Depressive symptoms in girls were more linked to the lack of protective family factors (9.9% of the explained variance in girls vs 5.5% in boys), while depressive symptoms in boys were more linked to the existence of harmful family factors (10.8% of the explained variance in boys vs 7.3% in girls).

**Conclusion:**

Family activities and the father's warmth and affection have a higher significance for girls than for boys, while destructive parental conflict and the mother's aggression and hostility are equally significant for both girls and boys. These results indicate the targets for family-based preventive and intervention programs for depression in adolescents.

Depression has been described as one of the most frequent internalized mental health problems experienced by the youth (1) and is associated with a great deal of personal distress and disruption (2). The incidence of depression significantly increases from childhood to adolescence from approximately 5% to 10%-20% (3). This rise may be attributed to the developmental process related to hormonal changes in puberty (4), a greater capacity for abstract thinking and rumination (5), increased psychological stress in developmental transition, or changing relations with parents and peers (6). Depressive symptoms may interfere with adolescents’ abilities to engage effectively in developmental tasks that may have an impact on their psychological health and well-being. In addition, depressed adolescents are at an increased risk of developing depressive disorders in adulthood (7).

The multiple pathways to depression are caused by the complex interplay among genetic, biological, cognitive, interpersonal, family, and environmental factors (2,8). Researchers have identified a large number of risk factors and, to a lesser degree, protective factors related to the manifestation of depression, with parental and family factors playing a big role in the former (8,9). The most often identified and analyzed family-related risk factors for child depression include parental rejection, harsh parental discipline, and lack of parental warmth and availability (10,11), as well as marital/family conflict, and parents’ stress and depression (12). The protective factors most often analyzed are supportive parental interaction, fostered through parental warmth, family cohesion, and common family activities (13,14).

The relationship between risk factors and the subsequent depression in adolescence varies by sex (15,16). While during preadolescence depression rates for boys and girls are approximately the same, during adolescence girls are more likely to develop more frequent and more severe depressive symptoms than their male counterparts (5). Sex may also moderate the relation with parents and marital conflicts as predictors of depression (5,16,17).

Although several studies suggest that family interactions play an important role in the development of depression (8), their results regarding the relevant parental or family factors contributing to it and the mechanisms through which they operate are inconsistent. Moreover, the respective influence of each parent on an adolescent’s social functioning and psychosocial adjustment is rarely specified, even more so when it comes to the role of the fathers. The multidimensional nature of protective and risk factors for depression and the possible differential contribution of relations with mother and father separately for men and women is stressed in recent research (17,18).

The aim of our study was to gain an insight into the relationship between protective and harmful family interactions and depressive symptoms among adolescent boys and girls through analysis of their perception of their mother’s and father’s relation toward them, family activities, and modalities of parental conflict resolution. We hypothesized that the specific family relations and each parent’s interaction were significantly correlated with depressive symptoms in adolescents, and that the positive family relations (closeness, warmth. and affection) had a more protective role for girls than for boys.

## Method

### Participants and procedure

The survey was conducted in January and February 2010 in all secondary schools (N = 30) in the Primorsko-Goranska County, Croatia. A representative proportional stratified sample of students was determined based on the initial data of the number of students enrolled in all classes in all county secondary schools (N = 10 770). The sample constitutes 13.20% of the population.

Random sampling of participants was conducted by research Randomizer service (*http://www.randomizer.org*). A total of 1422 students were included in the study, but only the data from those who lived with both parents (N = 1191), were processed (Table 1).

**Table 1:** Demographical characteristics of the students (n = 1191) in the study

Characteristic	No. (%) of students
**Sex:**	
male	574 (48.2)
female	617 (51.8)
**Type of school:**	
three-year vocational	159 (13.4)
four-year vocational	659 (55.3)
grammar school	373 (31.3)
**Student year:**	
first	259 (24.8)
second	316 (26.6)
third	325 (27.3)
fourth	253 (21.2)

The study was approved by the school’s founder (Primorsko-goranska County) as a competent administration authority. It was conducted in accordance with the Code of Ethics for Research with Children (19). Participants were informed that participation was voluntary and anonymous, and they gave their informed consent. No participant refused to take part in the study. A trained research team of psychology students administered the questionnaire to students (divided into groups of up to 25 students), who completed the survey material in about 45 minutes in their school setting.

### Measures

We measured adolescents’ depressive symptoms with the Depressive Symptoms Scale and a set of family variables with Family Rituals and Activities scale, Parental Conflict Strategies scales, and Parental Acceptance-rejection scale. The descriptive features of the measures are presented in Table 2, while their content is given in web extra material (web extra material 1).

**Table 2:** Descriptive statistics of the study variables and test of sex differences

	No. of items	Students (mean ± standard deviation)^†^		t	*P*
		boys (n = 574)	girls (n = 617)		
Depressive symptoms	11	24.24 ± 16.85	28.57 ± 16.65	4.46	<0.001
Protective family factors:					
family activities	11	48.88 ± 16.70	51.09 ± 16.83	2.28	0.023
PCS* – constructive mode	3	50.41 ± 23.73	57.52 ± 21.36	5.44	<0.001
mother – warmth and affection	6	72.06 ± 21.17	80.68 ± 19.02	7.40	<0.001
father – warmth and affection	6	65.85 ± 22.56	72.08 ± 22.03	4.82	<0.001
Harmful family factors:					
PCS – destructive mode	6	15.19 ± 15.93	17.05 ± 16.29	1.99	0.047
mother – aggression and hostility	5	31.00 ± 18.64	29.50 ± 18.26	1.40	0.161
father – aggression and hostility	5	28.21 ± 20.59	25.95 ± 20.82	1.88	0.060

*PCS – parental conflict strategies.

†The scores on all measures were linearly transformed to a standardized scale ranging from 0 (minimum score) to 100 (maximum score on the scale), with the aim to facilitate comparison between results on different variables.

### Adolescent depressive symptoms

Adolescents reported their depressive symptoms with the Depressive Symptoms Scale (DSS). DSS is a brief self-report measure which assesses the current level of an adolescent’s depressive symptomatology along dimensions of dysphoric mood, negative affect, negative self-evaluation, somatic complaints, loneliness, and suicidal ideation. Given that a large number of studies examined the depressiveness in adolescents using questionnaires created for adults (2), our scale was aimed to encompass the perceptions of depressive symptoms typical of the young.

The measure was developed as a part of a battery of short measures to be used for research of psychological adjustment of adolescents aged 14 to 18 years in Croatia. Since it is presented for the first time in this article, DSS is not validated in relation to clinically manifest depression. It only indicates the prevalence of depressive symptoms in general population of adolescents.

The DSS consists of 11 statements rated on a 4-point scale (0 – almost never; 1 – rarely; 2 – often; 3 – very often), indicating the frequency with which depressive symptoms are experienced.

Exploratory factor analysis was conducted to determine dimensionality and to justify the combining of items. Three factors were isolated: 1) interpersonal problems in the form of the feeling of abandonment and loneliness (eg, “I feel abandoned,” Cronbach α = 0.83); 2) general feeling of dejection and sadness with somatic symptoms of fatigue, exhaustion, dullness, sluggishness, nervousness, and anxiety (eg, “I am sad and depressed,” Cronbach α = 0.78); and 3) symptoms of existential crisis with suicidal thoughts (eg, “I had thoughts about committing suicide,” Cronbach α = 0.70). The intercorrelations of these three sets of items were relatively high and ranged between r = 0.49 and r = 0.67, which justifies their combination into a single measure of depressive symptoms. Cronbach α’s of the 11-item cumulative scale for boys and girls were 0.87 and 0.88, respectively.

DSS yields a total score that indicates the prevalence of some of the depressive symptoms in the general population of adolescents. The cut-off point for the high incidence of depressive symptoms in the sample was defined by the characteristics of the rating scale, where answers in the categories “often” and “very often” (2 and 3) were treated as indicators of a greater incidence of depressive symptoms.

### Family Rituals and Activities Scale

Family Rituals and Activities Scale consists of questions about family rituals and activities such as parties, holidays, trips, shows, movies, shopping, sports activities, board games, TV, and family discussions and walks (20). The scale consists of 11 statements rated on a 4-point scale from 0 (almost never) to 3 (very often), indicating the frequency of time spent with other members of family. The scale demonstrated high internal consistency (α = 0.85).

### Parental Conflict Strategies Scales

The two short scales of parental conflict strategies were used to assess adolescent’s perception of interparental conflicts. They measure two distinct approaches to dealing with a conflict: 1) constructive strategies or the use of rational discussion, argument, and reasoning (eg, “They try to listen to each other”), and 2) destructive strategies or the use of verbal and non-verbal acts that are harmful or threatening to another person (eg, “They threaten each other”). The scale was developed in line with conceptual ideas of Conflict Tactics Scale (21) designed to measure the use of reasoning, verbal aggression, and violence in the family. Constructive parental conflict strategy is a three-item measure (Cronbach α = 0.72), while the destructive conflict strategy is a six-item measure (Cronbach α = 0.83).

### Parental Acceptance-rejection Scale (PARS)

PARS is a short self-report measure developed from parental acceptance-rejection theoretical concepts (22). It assesses children’s reflection on their mother’s and father’s current behavior toward them. Two forms measuring mother’s and father’s behavior were used. They were virtually identical except for the sequence of items in the questionnaire. PARS is 11-item self-report measure assessing respondents’ perceptions of maternal and paternal warmth and affection (eg, “She/he openly expresses positive emotions and warmth”) and aggression and hostility (eg, “She/he punishes me even when I haven’t done anything wrong”). Parental warmth and affection is a six-item measure (Cronbach α for mother’s and for father’s behavior scale = 0.87). Parental aggression and hostility is a five-item measure (Cronbach α for mother’s behavior = 0.75, and for father’s behavior scale = 0.79). Individuals respond to items on a 4-point Likert-type scale from 0 (never) to 3 (very often). Scores on the parental warmth and affection and separately on parental aggression and hostility items were summed, producing two overall measures of perceived acceptance and perceived rejection.

### Data analysis

Cronbach α coefficients as a measure of internal consistency were calculated for all variables, while basic descriptive analysis was performed separately for boys and girls. The mean differences in study variables between boys and girls were tested by an independent samples *t*-test. Correlation analyses between variables were performed for boys and girls separately. The normality of variables was assessed by a normal probability plot. The distribution of results of the depressive symptoms scale was transformed by the square root transformation to improve its normality and linearity for regression analysis. Finally, a hierarchical multiple linear regression analysis was conducted to calculate the effects of family supportive and harmful interactions on depressive symptoms in both sexes. Statistical analyses were performed with the Statistica 9.1 statistical package (StatSoft Inc., Tulsa, OK, USA).

## Results

### Sex differences

The basic descriptive data of all variables with tests of difference between boys and girls are presented in Table 2. The results point to significant sex differences in depressive symptoms and the perception of some family interactions. Girls expressed significantly more depressive symptoms than boys, with 19.1% of them experiencing them often and very often, in contrast to 15.8% of boys, t(1,785) = 3.34. Girls’ assessment of the family relations when examined as a whole was significantly more positive than boys’, including family activities, constructive family conflict resolution, or father’s and mother’s warmth and affection.

### Correlation analysis

Correlation analyses between all variables were performed separately on boys and girls. The correlations are shown in Table 3. For both boys and girls, depressive symptoms were negatively related to supportive family factors, and positively to potentially harmful family factors.

**Table 3:** Bivariate correlations of variables for boys (n = 574) and girls (n = 617), shown respectively above and below the diagonal

Variable	1	2	3	4	5	6	7	8
1. Depressive symptoms scale	–	-0.09*	-0.04	-0.17^†^	-0.22^†^	0.29^†^	0.34^†^	0.26^†^
2. Family activities	-0.21^†^	–	0.11^†^	0.29^†^	0.33^†^	-0.14^†^	-0.11^†^	-0.07
3. PCS^‡^– constructive mode	-0.09*	0.26^†^	–	0.36^†^	0.30^†^	0.09*	0.00	-0.05
4. Mother – warmth and affection	-0.15^†^	0.43^†^	0.25^†^	–	0.54^†^	-0.12^†^	-0.22^†^	-0.15^†^
5. Father – warmth and affection	-0.30^†^	0.46^†^	0.21^†^	0.49^†^	–	-0.24^†^	-0.23^†^	-0.33^†^
6. PCS – destructive mode	0.32^†^	-0.25^†^	-0.12^†^	-0.22^†^	-0.40^†^	–	0.33^†^	0.35^†^
7. Mother – aggression and hostility	0.28^†^	-0.25^†^	-0.06	-0.47^†^	-0.26^†^	0.37^†^	–	0.57^†^
8. Father – aggression and hostility	0.28^†^	-0.19^†^	-0.12^†^	-0.18^†^	-0.48^†^	0.41^†^	0.44^†^	–

*P < 0.05.

†*P* < 0.01.

‡PCS – parental conflict strategies.

Relatively high intercorrelations of variables were observed with regard to both a positive family environment – common activities, constructive parental conflict resolution, and mother’s and father’s warmth/affection, and a negative family environment – inadequate forms of parental conflict resolution and negative mother/father relation.

### Hierarchical multiple regression analysis

To test the effects of all family variables on depressive symptoms, a hierarchical regression analysis was conducted with each family subscale as the predictor, using the two-steps model with two blocks of independent variables.

The first block of variables was composed of 4 “positive factor” variables, which potentially serve as family resources against depressiveness. The second block was composed of 3 potentially “harmful factors” for depressiveness. The results are shown in Table 4.

**Table 4:** Regression of depression on family variables

	Depressive symptoms											
	boys (N = 574)						girls (N = 617)					
	block 1			block 2			block 1			block 2		
	β	t	*P*	β	t	*P*	β	t	*P*	β	t	*P*
Step 1 – protective factors												
Family activities	-0.04	-0.95	0.344	-0.03	0.64	0.526	-0.11	-2.47	0.014	-0.09	2.06	0.039
PCS* – constructive mode	0.08	1.91	0.057	0.02	0.55	0.582	0.00	0.01	0.996	-0.01	-0.24	0.810
Mother – warmth and affection	-0.08	-1.62	0.105	-0.04	0.84	0.402	-0.01	-0.13	0.901	0.08	1.59	0.112
Father – warmth and affection	-0.18	-3.57	0.000	-0.08	1.63	0.103	-0.24	-5.25	0.000	-0.16	3.14	0.002
Step 2 – harmful factors												
PCS – destructive mode				0.18	4.28	0.000				0.15	3.38	0.001
Mother – negative relation				0.22	4.63	0.000				0.20	4.05	0.000
Father – negative relation				0.03	0.62	0.535				0.05	1.01	0.313
R^2^	0.055^†^			0.163^†^			0.099^†^			0.172^†^		
ΔR^2^				0.108^†^						0.073^†^		

*PCS – parental conflict strategies.

The analysis showed that the entire set of independent variables accounted for 16.3% of total variance of depressive symptoms for boys (F_7, 566_ = 15.76; *P* < 0.001; η^2^ = 0.163) and 17.2% for girls (F_7, 609_ = 18.09, α<0.001; η^2^ = 0.172). However, the effects and structure of relations between independent variables and depressive symptoms were different for boys and girls.

As shown in Table 4, protective factors, entered at Step 1, were significant in predicting depressive symptoms for both boys (F_4, 569_ = 8.32, *P* < 0.001; η^2^ = 0.055) and girls (F_4, 612_ = 16.90; *P* < 0.001; η^2^ = 0.099), but with a different predictive power and constellation of predictors. For boys, 5.5% of the depressive symptoms could be explained by protective factors, with fathers’ warmth and affection being the only significant predictor. In girls, 9.9% of the depressive symptoms could be explained by protective factors, with family activities and the fathers’ warmth and affection as two significant predictors (Table 4).

Harmful factors, entered at Step 2, significantly altered the constellation of the predictors of depressiveness, especially in boys’ sample. These newly-introduced variables accounted for an additional 10.8% of variance of depressive symptoms in boys and for 7.3% of variance in girls.

The most significant risk factor variables associated with depression in boys and girls were the mothers’ aggression and hostility and aggressive parental conflict resolution. In Step 2, practically none of the positive family factors played a significant protective role for depressiveness in boys’ sample, while the fathers’ warmth and affection still had a significant predictive power in girls’ sample. A positive family climate was more relevant for the protection from depressiveness in girls than in boys, while a negative family environment played an important role in depressiveness in both sexes.

## Discussion

We found the incidence of depressive symptoms in the adolescent sample to be relatively high, 19.1% the girls and 15.8% of the boys experienced depressive symptoms often or very often. The sex difference in the prevalence of depressive symptoms found in this study is in accordance with the results already demonstrated by other studies (23-28).

We confirmed our hypothesis that family relations and the interaction with each parent significantly contributed to adolescents’ depressive symptoms (they accounted for 17.2% of the variance in girls’ depressive symptoms, and for 16.3% in boys’), with different constellations of predictors in boys’ and girls’ sample. This is in contrast with previous studies (29) that showed greater sex difference.

Our results show also that different family variables predict depressive symptoms in boys and girls. While parental conflict and mother’s aggression and hostility were significant predictors of depressive symptoms in both boys and girls, family activities and father’s warmth and affection played a significant role only for girls. Despite the finding that during adolescence same-sex attachments to parents are more important than different-sex attachment relationships (30), we found that a warm relation with the mother was important for both boys and girls, while a warm relation with the father was more important for the girls. Our results suggest that the presence of an involved and nurturing father figure provides additional protection against health-risk behavior for girls, which is in line with other studies (13,31,32). A warm relation with parents may play a protective role because it communicates to children that the family will continue to be a source of stability and support to them, despite their sense of disrupted family relationships (33).

Despite the fact that the relation with both parents significantly correlated with depressive symptoms (Table 3), when their joint effect is observed, only the rejection by the mother emerged systematically as a predictor, especially in the context of an aggressive conflict resolution pattern. The importance of mother’s rejection is in accordance with other studies that stress the strong relation between parental hostility and child depression (10,34,11). The results of our study are in line with studies that have shown how elevated levels of conflicted interactions and poor-quality family relations (degree of trust, warmth, fun, and togetherness) contribute to the development of depressive symptoms (35-38). The significance of both the relation with parents and family conflict resolution may be explained through the family system theory according to which different family subsystems (marital, parent-child, and sibling relationships) mutually influence each other (39). Emotions or behavior in a specific family subsystem may influence other subsystems within the family. According to the spillover hypothesis, hostile relationships between parents are transferred to the parent-child relationship (40). We also found that the perceived hostile exchanges between parents were connected with the perceived hostility toward children (Table 3). The emotional security theory stresses that interparental conflict may decrease the likelihood of establishing positive relationships with children and increase the children’s insecurity and vulnerability to mental illnesses (41-44).

Our study confirmed the protective role of family activities as an aspect of family cohesion only for girls. For girls, emotional bonding and feeling of closeness in the family appears to be a buffer against the development of internalizing disorders (45,46). A possible explanation for this association may be the girls’ orientation to social relations and higher levels of emotional closeness to significant others (47). Girls who perceive a lower level of family activities and an emotional distance from parents may develop a feeling of unworthiness, which in turn results in depressive symptoms or some other behavioral problem, such as substance use (48). In contrast to the views about the adolescents’ becoming autonomous through emotional distancing from parents, this study confirms the parents’ role in the emotional development of both boys and girls. Although the risks of and protection from depressive symptoms were derived from multiple fields of adolescent experience and engagement, the experience within the family plays a pre-eminent role (49,50). Our study revealed that the interaction between the family relationships and depressive symptoms was moderated by the child’s and parent’s sex. This study stresses the importance of integrating perceptions of family conflict with sex-specific risk for adolescent depression.

This study confirms the importance of developing treatment strategies in preventive and public health through family-based prevention and intervention programs for depression (51,32,49). These programs are supposed to include different approaches thorough parental education about different developmental paths for adolescent boys and girls and developing skills in interpersonal problem solving. Advising families on how to reduce conflict and enhance family warmth, promotion of common pleasurable family activities, and stressing the importance of the fathers’ warmth and affection as a significant protective aspect in parent-adolescent relationship should be pointed out in interventions with families.

One of the strengths of this study is that it examined depressive symptoms in a representative and large community-based sample of high school students who live with both parents. We applied a new scale for measuring depressive symptoms in adolescence, which has shown high reliability but still has to be validated in future studies.

The major limitation may be the fact that all data were obtained from a single source as self-report measures with common method variance. A cross-sectional and correlational study does not allow a cause-and-effect conclusion. Adolescents with more depressive symptoms are likely to have a biased perception of their family relations or even, according to some recent longitudinal studies, induce negative mood in others, which is why their parents stop supporting them (16).

The modest percent of variance in depressive symptoms explained by family variables in our study indicates that relevant data should be derived from multiple fields of adolescent experience and engagement. Protective factors that can additionally explain mental adjustment problems can be related to a positive self-concept, better relations with peers, and better school connectedness (49). The future research should examine the interactions between sex, vulnerability factors, and stressors in order to determine if girls are more susceptible to certain vulnerability factors than boys, specifically in the face of stressful life events. Studying the differences in risk and protective factors during the adolescent period through longitudinal research may additionally explain the sex differences in the development of adolescents’ depressive symptoms.
