# Supplementary material for: Sex differences in patterns of relations between family interactions and depressive symptoms in adolescents
Source: Croat Med J. 2011 Aug;52(4):469–77. doi: 10.3325/cmj.2011.52.469 (PMC3160708; doi:10.3325/cmj.2011.52.469)
Supplement: Supplementary material 1 [file CroatMedJ_52_s001.pdf]

Appendix
Content of scales

Depressive symptoms ***(Cronbach alpha=.88)***

Depressiveness - Somatic Symptoms

I am sad and depressed

I feel tired and exhausted

I feel sluggish and slow

I am nervous and anxious

Loneliness

I don't feel that I belong to the world I live in

Nobody understands me

I have no one to turn to

I feel abandoned

Existential crises

Life is mainly very boring

I had thoughts about committing suicide

I think I am not worth anything

Family activities ***(Cronbach alpha=.85)***

We attend sports events or do sports activities

We go to the theatre or to the cinema

We play games

We go for walks

We study and write homework together

We go for family trips and excursions

We watch TV

We eat lunch or dinner together

We talk and have discussions

We spend summer/winter holidays together

We go shopping together

Parental warmth and affection ***(Mother - Cronbach alpha=.87; Father - Cronbach alpha=.87)***

I can count on her/his help

She/he has understanding for my feelings

She/he is honest

I always feel protected around her/him

She/he openly expresses positive emotions and warmth

She/he notices when I’m upset about something

Parental aggression and hostility ***(Mother - Cronbach alpha=.75; Father - Cronbach alpha=.79)***

She/he constantly has objections to my actions

She/he is not satisfied with whatever I do

She/he yells at me

She/he punishes me even when I haven’t done anything wrong

She/he doesn’t allow me to do things which are allowed to my peers

Parental conflict strategy – Constructive mode ***(Cronbach alpha=.72)***

They negotiate

They give explanations for their point of view

They try to listen to each other

Parental conflict strategy – Destructive mode ***(Cronbach alpha=.83)***

They threaten each other

They insult each other

They yell at each other

They try to win me over to their side

They get into physical conflict

They question me about what the other does/thinks/says
